# *PDK1* and *HR46* Gene Homologs Tie Social Behavior to Ovary Signals

**DOI:** 10.1371/journal.pone.0004899

**Published:** 2009-04-02

**Authors:** Ying Wang, Gro V. Amdam, Olav Rueppell, Megan A. Wallrichs, M. Kim Fondrk, Osman Kaftanoglu, Robert E. Page

**Affiliations:** 1 School of Life Sciences, Arizona State University, Tempe, Arizona, United States of America; 2 Department of Chemistry, Biotechnology and Food Science, University of Life Sciences, Aas, Norway; 3 Department of Biology, University of North Carolina–Greensboro, Greensboro, North Carolina, United States of America; Pennsylvania State University, United States of America

## Abstract

The genetic basis of division of labor in social insects is a central question in evolutionary and behavioral biology. The honey bee is a model for studying evolutionary behavioral genetics because of its well characterized age-correlated division of labor. After an initial period of within-nest tasks, 2–3 week-old worker bees begin foraging outside the nest. Individuals often specialize by biasing their foraging efforts toward collecting pollen or nectar. Efforts to explain the origins of foraging specialization suggest that division of labor between nectar and pollen foraging specialists is influenced by genes with effects on reproductive physiology. Quantitative trait loci (QTL) mapping of foraging behavior also reveals candidate genes for reproductive traits. Here, we address the linkage of reproductive anatomy to behavior, using backcross QTL analysis, behavioral and anatomical phenotyping, candidate gene expression studies, and backcross confirmation of gene-to-anatomical trait associations. Our data show for the first time that the activity of two positional candidate genes for behavior, *PDK1* and *HR46*, have direct genetic relationships to ovary size, a central reproductive trait that correlates with the nectar and pollen foraging bias of workers. These findings implicate two genes that were not known previously to influence complex social behavior. Also, they outline how selection may have acted on gene networks that affect reproductive resource allocation and behavior to facilitate the evolution of social foraging in honey bees.

## Introduction

The evolution of social behavior in insects is key to one of the most successful transitions in the history of life [1], though much is to be discovered about its evolution from solitary origins. Complex societies, like those of the honey bees, consist of a primary female reproductive, the queen, and thousands of facultatively-sterile female workers [2]. Natural selection operates on populations and results in changes in function and expression for genes expressed in the normally non-reproductive workers, effecting morphological, physiological, and behavioral differentiation resulting in division of labor, the hallmark feature of social insects. Evolutionary changes in social structure must be a result of changes in development of individuals, presenting one of the most important questions for understanding social evolution: how were developmental ground plans of solitary ancestors altered to produce social systems?

The honey bee society is a model system to study the developmental evolution and current regulation of complex social structures. They largely consist of two female castes: the reproductive queens and functionally sterile workers, which perform all other colony tasks in an age-associated manner [3]. The younger workers perform tasks in the nest that change with age and the needs of the colony. The youngest bees clean cells. Then as they mature they normally progresses to feeding larvae, nest construction and food processing, then in about the second or third week of adult life, transition to foraging outside the nest, primarily for pollen and nectar. Amdam et al. [4] proposed that foraging division of labor in honey bees is influenced by reproductive gene networks that are linked to behavior in solitary insects. This hypothesis was derived from the Ovarian Ground Plan Hypothesis of West Eberhard [5], [6] and consequently named the Reproductive Ground Plan Hypothesis (RGPH). The central prediction of the RGPH is that worker reproductive anatomy and physiology is linked to biases in foraging behavior. Many insects forage for protein in order to fully activate their ovaries and produce eggs. Nest-provisioning insects also hoard food-items high in protein to support the developing young [4]. Honey bee workers do not normally lay eggs, but wild-type bees (unselected commercial stocks) with large ovarian structures (more ovariole filaments in each ovary) are more likely to collect pollen, a source of protein, to express *vitellogenin* (*Vg*, a yolk protein precursor) mRNA at higher levels as young adults, and to initiate foraging earlier in life than workers with fewer ovarioles [7], [8]. Bi-directional colony-level selection on pollen-hoarding, likewise, resulted in high strain worker bees with more ovarioles, foraging bias toward pollen, increased levels of Vg in young adults, and earlier foraging onset than low strain bees [9]. In honey bees, Vg synthesis is turned on immediately prior to adult emergence in response to signaling by ecdysteroids and juvenile hormone, which are insect hormones that normally govern reproductive events in the mature adult stage [10]. Yet instead of egg-laying, workers express maternal care behavior toward siblings, including food provisioning and pollen hoarding [4].

The associations between insect reproductive signaling and behavior can have persisted through the evolutionary process toward sociality because their genetic bases are largely congruent. At the phenotypic level, correlative links between worker ovary size and foraging division of labor were confirmed repeatedly in wild type and selected pollen-hoarding strains (see above, [7]), and effects of Vg on foraging behavior was verified by *Vg* knockdown [11]. However, it has not been tested if the correlation of ovary size and behavior are due to direct genetic relationships, as predicted by the RGPH.

The high and low pollen-hoarding strains [9] represent the most comprehensively studied model of honey bee foraging behavior [Bibr pone.0004899-Page2] [see reviews in ref. 12 and 13]. Divergent artificial selection has significantly altered their social structure associated with foraging. Genetic analyses have revealed four major quantitative trait loci (QTL), *pln1*–*4*, with broad pleiotropic and epistatic effects [14]–[17]. The mapped QTL regions are located on chromosome 1 (*pln2*: 16.3–19.3 Mb; *pln3*: 7.9–9.4 Mb with an approximate minimum recombination distance of 120 cM) and chromosome 13 (*pln1*: 5.2–7.1 Mb; *pln4*: 8.9–9.1 Mb with an approximate recombination distance of 100 cM). Thus all QTL are genetically independent. They are enriched with candidate genes belonging to the insulin/insulin like signaling (IIS) pathway, including *PAR 3* (bazooka, GB10346), *PI3K* (phosphoinositide 3-kinase, GB17429), *PDK1* (phosphoinositide-dependent kinase-1, GB15780), and *IRS* (insulin receptor substrate, GB11037), that can govern resource allocation to reproduction and life-history progression [13]. Also, the QTL architecture includes a nuclear hormone receptor (NHR) homolog, *HR46* (hormone receptor-like in 46, GB10650; referred to before as *dHR3* in *Drosophila*). *HR46* may affect ovary size by acting on *βFTZ-*F1 (an orphan NHR) to change organ morphology during development [18], [19]. The pleiotropic nature of the *pln* QTL hierarchy [14]–[17] and the inferred molecular functions of the underlying candidate genes suggest that these loci represent central nodes (switches) in genetic modules that governed the reproductive phenotype of solitary ancestors before being co-opted as ontogenetic regulators of social insect phenotypic plasticity [see a review in ref. 13].

Here we use the high and low pollen-hoarding strains to demonstrate: *i)* that two of the four QTL for foraging behavior, *pln2* and *pln3* , have direct genetic effects on ovary size; *ii)* that ovary size and foraging behavior are genetically correlated; *iii)* that two candidate genes, *HR46* (*pln2*) and *PDK1* (*pln3*), show significantly different tissue-specific expression patterns between bees with different social behavior; and *iv)* that the same tissue-specific patterns demonstrate a significant genetic correlation with ovary size. Collectively, the linkage of the *pln2* and *pln3* genome regions to foraging behavior and ovary size, the genetic linkage of candidate genes within them to ovary size, and the genetic linkage of ovary size to foraging behavior provide evidence for the central prediction of the RGPH: honey bee foraging division of labor shares a common genetic basis with a reproductive trait.

## Results

### Effects of pln QTL on ovary size

To test for linkage between the behavioral *pln* QTL regions and ovary size, we produced backcrosses of the high and low pollen-hoarding strains. Backcross designs are powerful genetic tools that allow meiotic recombination to sever trait associations that are not genetically linked. Ovary size, measured as the total number of ovariole filaments in both ovaries, was 10.0±1.0 (s.e.m.) and 3.8±0.5, in the parental high and low strains (n = 20), respectively, with strain explaining 33% of the total phenotypic variation. In the resulting backcrosses, the two ovaries were correlated in size within workers (high backcross ‘HBC’: R = 0.63, n = 392, p<0.001; low backcross ‘LBC’: R = 0.60, n = 393, p<0.001). To account for observed intra-individual variation in ovary size, the smaller and the bigger ovary were analyzed as two separate variables to partition their contribution to the main variable, total ovariole number (see below). The resulting hybrids had significantly fewer ovarioles (4.1±0.6) than the high strain and were statistically indistinguishable from the low strain (Dunnett's C post-hoc tests). The HBC (8.0±0.2) was not significantly different from the high strain, in contrast to the LBC (5.7±0.2) that formed a statistically homogeneous subset with the hybrid and the low strain parent. Similar directional dominance for the low strain phenotype has been shown repeatedly [14]–[17].

**Figure 1:**
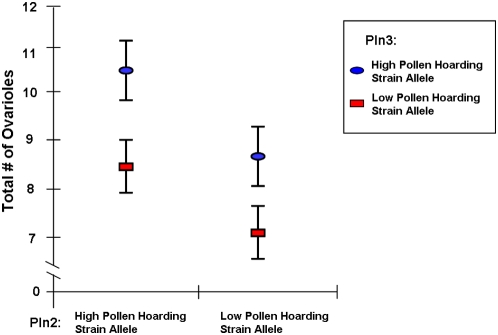
Effects of *pln*-QTL on ovary size. Behavioral *pln2* and *pln3* QTL showed direct, additive genetic effects on the total number of worker ovarioles (mean±s.e.m.) in a high strain backcross between the selected pollen-hoarding strains.

In the HBC population, markers for *pln* QTL showed a genetic effect on the total number of ovarioles. The full factorial ANOVA indicated a direct effect of *pln2* (F_(1,140)_ = 4.3, p = 0.040) and *pln3* (F_(1,140)_ = 5.1, p = 0.025), as well as an interaction between all four *pln*-QTL (F_(1,140)_ = 3.9, p = 0.050). The main effects were confirmed by non-parametric analyses (*pln2*: Mann-Whitney U = 3235.5, n = 178, p = 0.037; *pln3*: U = 3152.0, n = 175, p = 0.043). In both cases, the allele from the high pollen-hoarding line increased the number of ovarioles by approximately 1.3 ovarioles (Fig. 1). The corresponding two-factorial ANOVA model was significant overall (F_(3,160)_ = 3.1, p = 0.030) and reconfirmed the two direct effects of *pln2* (F_(1,160)_ = 4.7, p = 0.032) and *pln3* (F_(1,160)_ = 5.1, p = 0.026) without a significant interaction term (F_(1,160)_ = 0.04, p = 0.846).

Analyzed separately, the minimum ovariole number reconfirmed the direct influences of *pln2* (F_(1,140)_ = 4.5, p = 0.036) and *pln3* (F_(1,140)_ = 6.8, p = 0.010) and also showed significant interaction terms of *pln1*×*pln2*×*pln3*: (F_(1,140)_ = 5.4, p = 0.022) and among all four QTL (F_(1,140)_ = 5.2, p = 0.024). Non-parametric tests validated the direct results of *pln3* (Mann-Whitney U = 3055.0, n = 175, p = 0.019 but not *pln2* (Mann-Whitney U = 3347.0, n = 178, p = 0.076). The two-factorial ANOVA model, restricted to *pln2* and *pln3* was significant overall (F_(3,160)_ = 3.6, p = 0.014), with both direct effects significant (*pln2*: F_(1,160)_ = 4.9, p = 0.029; *pln3*: (F_(1,160)_ = 6.6, p = 0.011), indicating no interaction between the two (F_(1,160)_ = 0.03, p = 0.873). Maximum ovariole number was less influenced by the *pln*-QTL markers and showed no significant effects in the full factorial analysis. Analyzed separately, the only significant effect was *pln2* (Mann-Whitney U = 3253.0, n = 178, p = 0.041).

In the LBC, neither single nor multi-factorial analyses revealed significant genetic effects on total, minimum, or maximum worker ovariole number. This was expected due to the directional dominance of the low alleles (see above, [14]–[17]).

### Associations between ovary size and behavior

Next, we performed a behavioral verification test to ensure that ovary size was linked to foraging division of labor, and that behavioral traits were correlated with each other in backcross workers. These traits covary in pollen-hoarding strains and in wild-type bees, and are part of the honey bee pollen-hoarding syndrome [see a review in ref. 13]. For this backcross, the mean ovariole number (±s.e.m.) was 13.2±1.0 (n = 19) and 8.1±0.8 (n = 30) for the parental high and low strain sources, respectively. The hybrid queen source had 10.8±0.9 (n = 20) ovarioles on average, whereas four HBC and LBC had overall averages 10.4±0.2 and 7.0±0.8. Based on its high phenotypic variability and its representative average, we chose one HBC (W85) for testing individual phenotypic linkage of ovary size to behavior, and of different behavioral traits to each other.

**Figure 2:**
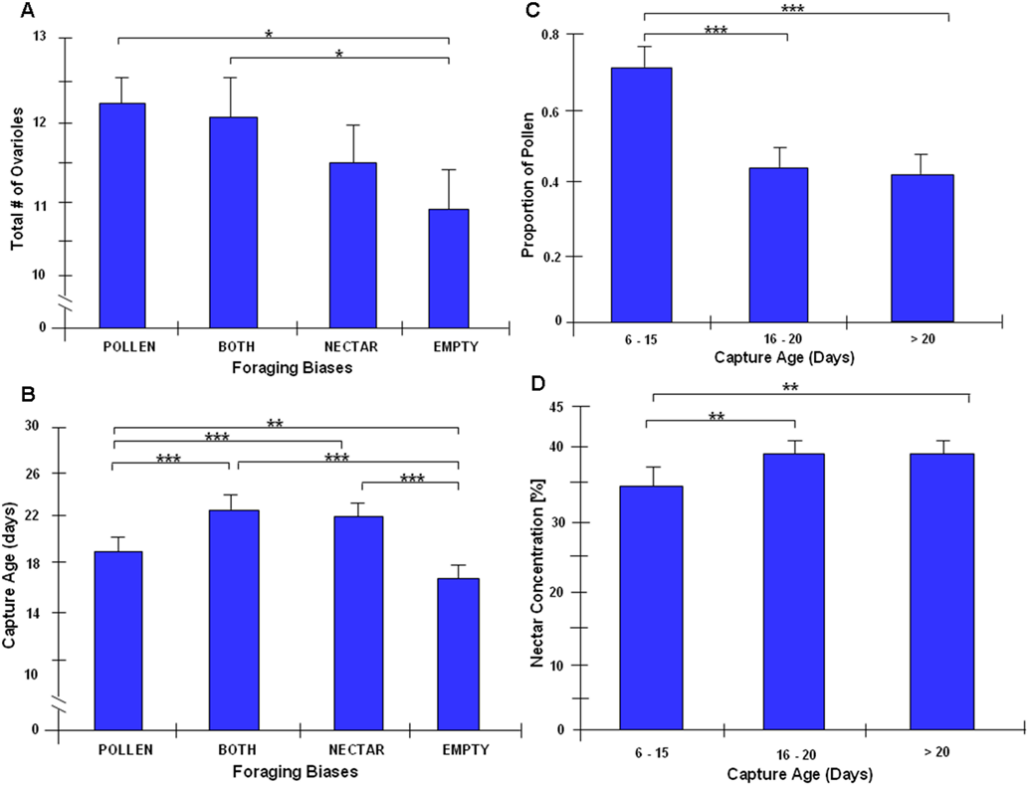
Associations between ovary size and behavior. A: Total ovariole number (mean±s.e.m) in different foraging behavior groups derived from a backcross of high and low pollen-hoarding strains (BOTH = pollen and nectar; EMPTY = no measurable pollen and nectar; POLLEN = pollen and NECTAR = nectar). B: The capture age (first foraging age) (mean±s.e.m) in different behavior groups. C, D: The pollen load proportion and nectar load concentration of the same bees divided by different capture age groups. *, <0.05; **, <0.01; ***, <0.001.

Returning bees were divided into four groups based on behavioral performance [20]: i) EMPTY bees returned with no measurable pollen or nectar (n = 48); ii) POLLEN foragers returned with pollen loads weighing more than 0.0002 g (n = 244); iii) NECTAR foragers returned with liquid crop content ≥0.002 g and with sucrose concentrations ≥10% (n = 124); and iv) BOTH foragers, which met the criteria for both POLLEN and NECTAR foragers (n = 129). POLLEN foragers and BOTH foragers had significantly more ovarioles than EMPTY bees (Student t-Test, t = 2.26, df = 80, p = 0.027; t = −1.98, df = 92, p = 0.050) (Fig. 2A), consistent with earlier results [7]. Foraging preference, in turn, was correlated with nectar concentration and with age at foraging onset. We divided the data into three groups on the basis of capture age (first foraging day): capture age 6–15 days, 16–20 days, and 21 days or older. There are significant differences in capture age between the behavior groups (POLLEN, BOTH, NECTAR and EMPTY; one-way ANOVA: F_(3, 541)_ = 14.49, n = 545, p<0.0001; Fig. 2B). Workers that initiated foraging later in life were more likely to forage for nectar (One-way ANOVA: F_(2, 508)_ = 17.46, n = 512, p<0.001) and collect higher nectar concentrations (One-way ANOVA: F_(2, 236)_ = 4.98, n = 240, p = 0.008; Fig. 2C, 2D). These trait-associations are consistent with the pollen-hoarding syndrome of honey bees [12].

### Patterns of pln candidate gene expression level

We used the parental strains to determine expression patterns of candidate genes for behavior after using backcrosses between high and low pollen-hoarding strains to establish genetic links between the behavioral QTL *pln2* and *pln3* and ovary size, and between ovary size and foraging behavior. A list for these genes was published previously [21]. Gene transcript was quantified by real-time RT-PCR (qRT-PCR) for third instar larvae, young adults (newly emerged bees), and foragers using two sources of each strain (see Materials and Methods). qRT-PCR has a low technical error rate and is a sensitive method for detection of gene transcript abundance, allowing for a sample size of 12 to test the expression differences between high and low strains. The data were log-transformed to approximate normality and analyzed by Student t-tests. Results were confirmed by non-parametric Mann-Whitney U tests on untransformed data (results not shown). *Actin* was used as housekeeper gene [22], [23], but because *Actin* expression can vary between life-stages [24], data were not used for inference between sample groups of different age.

**Figure 3:**
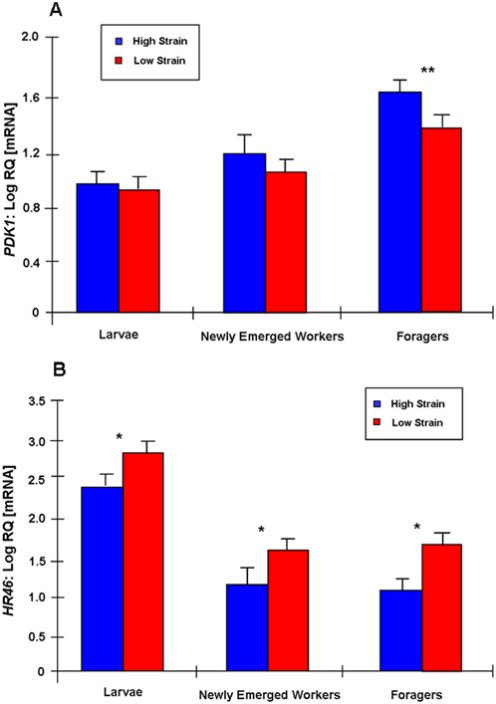
Comparison of *pln* candidate gene expression in pollen-hoarding strain bees. Log transformed mRNA levels (mean±s.e.m., relative quantities, n = 12) in the abdominal fat body of high (blue bars) and low strain bees (red bars). A: *PDK1* is expressed at higher levels in high strain foragers than in low strain foragers. B: *HR46* is expressed at higher levels in larvae, newly emerged workers and foragers of the low strain in comparison to the low strain. *, <0.05; **, <0.01; ***, <0.001.

*PDK1* (*pln* 3) expression was not significantly different between strains in larvae, in adult brain [supporting information (SI) Fig. S1 and Table S1] or in the fat body (analogous to liver and adipose tissue) of newly-emerged bees (t = 0.26, df = 19, p = 0.795; t = 1.18, df = 15, p = 0.258). In foragers, however, the high strain sources had significantly higher fat body mRNA levels of *PDK1* (0.32-fold) than the foragers of the low strain (t = 3.37, df = 20, p = 0.003) (Fig. 3A).

*HR46* (*pln 2*) expression differed significantly between high and low strains in all stages of development (t = −2.78, df = 15, p = 0.014; t = −2.92, df = 21, p = 0.008; t = −4.15, df = 11, p = 0.002, for larvae, newly emerged workers, and foragers, respectively). In the adults, the difference in *HR46* transcript level was specific to fat body, with levels in larvae, newly emerged bees, and foragers being higher in the low strain by approximately 1, 2, and 8-fold in untransformed data, respectively (Fig. 3B; SI Fig. S2 and Table S1).

For the other central candidate genes [21], *PAR3* (*pln 1*) (SI Fig. S3), *PI3K* (*pln 3*) (SI Fig. S4), and *IRS* (*pln 4*) (SI Fig. S5), mRNA levels in larvae (SI), the abdomen (SI) and brain (not shown) did not differ between the high and low strain sources (p>0.05).

### Associations between candidate gene expression level, ovary size and behavior

**Figure 4:**
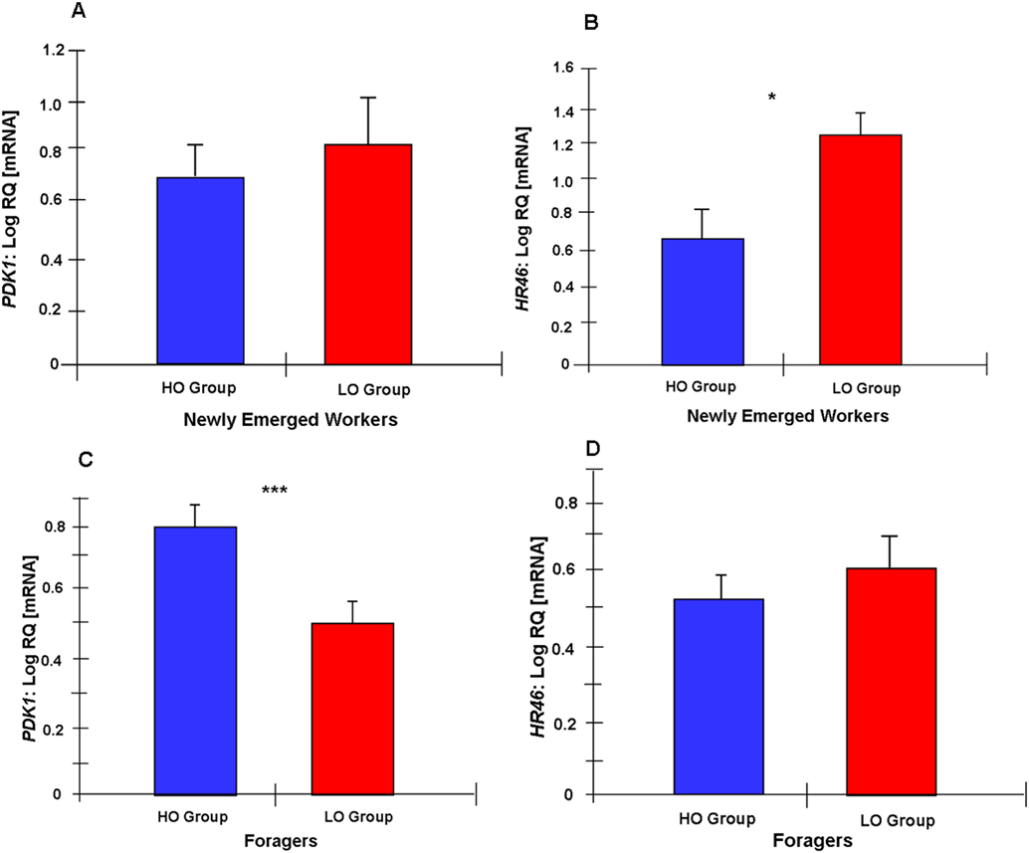
Associations between gene expression, ovary size, and behavior. Log transformed mRNA levels (mean±s.e.m., relative quantities, n = 24) of candidate genes in the abdominal fat body of newly emerged workers and foragers derived from a backcross of high and low pollen-hoarding strain bees. Workers are divided into groups with extremely low (LO) or high (HO) ovariole numbers. A: *PDK1* shows no significant difference between groups of newly emerged bees with different ovariole number. B: *HR46* mRNA levels are significantly higher in newly emerged bees with low ovariole number compared to the group with high ovariole number. C: *PDK1* gene expression is significantly higher in foragers with high ovariole number compared to the group with low ovariole number. D: *HR46* shows no significant difference in the foragers. *, <0.05; **, <0.01; ***, <0.001.

To test the genetic linkage between expression of *PDK1*, *HR46*, reproductive anatomy, and social foraging, we collected 697 newly emerged backcross workers (see Materials and Methods). After determining the ovariole number of the bees, we chose a high (large) ovary group (HO, n = 24) with an average ovariole number (mean±s.e.m.) of 24.7±0.6, and a low (small) ovary group (LO, n = 24) with 4.2±0.2 ovarioles. Individual gene transcript levels were determined for fat body tissue using qRT-PCR. Consistent with the results from the parental high and low strains (see above), we found that *HR46* was expressed at a significantly higher level (1.2-fold) in the LO group compared to HO group (t = 2.62, df = 42, p = 0.012; Fig. 4B). *PDK1*, also as before, was equally expressed in newly emerged workers (t = 0.66, df = 20, p = 0.52; Fig. 4A).

Next, we collected mature backcross foragers as they returned from the field (n = 571). As before, bees were dissected and divided into a large ovary group (HO, n = 24) that had 20.0±0.4 ovarioles and a small ovary group (LO, n = 24) with 7.9±0.4 ovarioles. We found that *PDK1* was expressed at significantly higher levels in the HO forager group compared to the LO forager group (t = 3.47, df = 44, p = 0.001; Fig. 4C), again mirroring the results from the parental strains (above). In parallel, *HR46* was equally expressed between the groups (t = 1.06, df = 43, p = 0.294; Fig. 4D).

## Discussion

We confirmed the link between the ovariole number and foraging behavior in backcross foragers derived from high and low pollen-hoarding strains. POLLEN foragers and BOTH foragers collecting both pollen and nectar have more ovarioles than the foragers returning empty. This result is consistent with those reviewed by Page et al. [25] that also show differences between bees returning with empty loads and those that carry pollen. Our results show the expected relationships of ovariole number to behavior (POLLEN>BOTH>NECTAR>EMPTY), but our sample sizes were sufficient only to distinguish between the most extreme phenotypes.

We found significant differences in capture age between the behavioral groups of the backcross, again confirming the relationships between foraging onset and food collection biases. POLLEN foragers initiated foraging at significantly younger ages than BOTH and NECTAR groups. These results are consistent with data obtained previously from high- and low-strain and wild-type bees [7], [9]. New to our study was the observation that EMPTY bees, which had the smallest ovaries on average (Fig. 2A), were captured significantly earlier than the behavioral groups that returned with loads of pollen and/or nectar. On the one hand, EMPTY bees can be unsuccessful foragers, and based on a simplified linear model: “the smaller the ovary the later the foraging onset”, we would have predicted higher capture ages in this group. On the other hand, in a recent study the wild-type bees with the smallest ovaries showed the strongest suppression of *Vg* mRNA [8], and experimental reduction of *Vg* expression by RNA interference mediated gene knockdown caused bees to forage earlier in life than handling control [11]. Thus, the trait associations of EMPTY bees are difficult to analyze without further experiments that take non-linear dynamics into account. Overall, however, our results clearly demonstrate, as with other studies, that ovary size (ovariole number) is linked to foraging age and behavior [26], [27], [28].

The pollen-hoarding syndrome of the honey bee consists of a common set of correlated behavioral phenotypes that include sucrose responsiveness, foraging preference, and the age at which bees initiate foraging [29], [30]. The syndrome is influenced by four major QTL with pleiotropic effects on behavior [16], [17], [31], [32]. The results presented here demonstrate that the genetic architecture of the pollen-hoarding syndrome also affects ovariole number [7]. *Pln2* and *pln3* demonstrate direct genetic effects on the ovary in worker bees while genetic interaction effects were found among all four *pln* QTL.

Despite the significant higher-order interaction characteristic of complex signaling cascades [21], the main effects of *pln2* and *pln3* on worker ovary size are additive, which could be explained by their involvement in two parallel, convergent genetic pathways (see below). This interpretation, and the roles of *pln2* and *pln3* as main links between ovariole number and the pollen-hoarding syndrome, is supported by the consistent, complimentary gene expression differences in *HR46* and *PDK1*, which are located in these QTL regions, respectively.

Of the five genes investigated based on a previously published candidate gene list [21], *PDK1* and *HR46* showed consistent, tissue-specific expression differences between pollen-hoarding strains and between backcross workers with large or small ovaries. These results from a backcross directly connect the pollen-hoarding syndrome to ovary size and gene expression patterns in workers because trait associations that are not mechanistically linked are severed by meiotic recombination through the experimental design. Thus, we show here a direct genetic linkage between social behavior, ovary size, and expression of *PDK1* and *HR46* in worker honey bees. Our results provide comprehensive support for the RGPH of Amdam et al. [4], and are consistent with central roles of IIS and ecdysteroid cascades in the architecture of the reproductive ground plan (see below).

### Role of IIS and PDK1 in foraging behavior strategy

The IIS pathway is convergent but largely independent of the ecdysteroid cascade [33]. It plays important roles in regulating insect life span, reproductive state, growth, and metabolism [34], [35]. *PDK1*, a candidate gene for *pln3*, is a kinase with important roles in IIS pathway function [36] as a down-stream up-regulator acting through *PKB* [37]. Fine-scale QTL mapping in *Drosophila* suggests that IIS may be responsible for variation in ovary size [38], but less is known about effects on behavior. Neuronal IIS, including *PDK1* function, can affect chemotaxis behavior and learning [39], a trait that varies between high and low pollen-hoarding strains [see refs. 12]–[13 for recent reviews]. Yet, our expression results suggest that the associations of *PDK1* and phenotype are neither developmental nor directly neuronal because *PDK1* mRNA levels were not different in the larvae, newly emerged stages, or the brains of high, low and backcross bees. Instead, our results point toward a regulatory system in which the capacity for *PDK1* up-regulation in forager fat body is conditional on ovary size. This hypothesis supports the idea that ovarian signaling is directly involved in affecting the physiology and behavior of foraging bees [40].

Despite the equal transcript levels of *PDK1* between larvae of high and low pollen-hoarding strain bees, *pln3* did significantly affect ovary size in the backcross. Ovary size is determined in larvae [41], and thus our results appear to exclude *PDK1* as a causal to ovary size. This outcome, however, can be explained by several factors: i) our transcriptional profiling is blind to additional structural variation in *PDK1* that could influence kinase activity, ii) our *PDK1* transcript profiling amplifies sequence that is common to the full set of *PDK1* isoforms (Wang, unpublished data), and thus it is insensitive to cis-regulated changes in the relative abundance of different *PDK1* isoforms; iii) a yet untested gene in *pln3* is responsible for the effect on ovary size. Interestingly, the ecdysone-related gene Cytochrome P450 (*Cyp307a1*), a regulator of ecdysone synthesis, is also located in *pln3* [42]. This gene could take part in the hormonal cascade that affects ovary size during development [43]. At the same time, *Cyp307a1* may influence *PDK1* expression, but further studies are needed to clarify these relationships.

### Role of Ecdysone cascade and HR46 in foraging behavior strategy

In the fly, *HR46* (or *dHR3*) is an early ecdysone-inducible nuclear hormone receptor. Peak expression coincides with ecdysteroid release in larvae, pupae, and adults [44], [45], and the gene is essential for normal molt progression and nervous system development. Accordingly, we confirm variation in *HR46* expression in groups that previously were characterized by changing hormone levels (larval and newly emerged adult stages) [10], [43]. The finding that transcript levels are not different between backcross foragers with diverging ovary sizes likewise fit the observation that ecdysteroid signaling, in general, is very low in mature adult honey bees [46].

In *Drosophila*, it was demonstrated that *dHR3* and *betaFTZ*-F1 act together to mediate the ecdysone response in larval and prepupal stages [47]. *dHR3* is an essential regulator of *betaFTZ*-F1, which can affect apoptosis during development [18], [19], [48]; as an example, *betaFTZ*-F1 influences salivary gland apoptosis during metamorphosis in the fly [49], [50]. Honey bee ovary size is also influence by an apoptotic cascade [41], [51], and we propose that *HR46* is one mediator of this process. This hypothesis presents the first candidate gene with regulatory potential to mediate the link between reproductive anatomy (ovary size) and honey bee worker behavior.

### Conclusions

**Figure 5:**
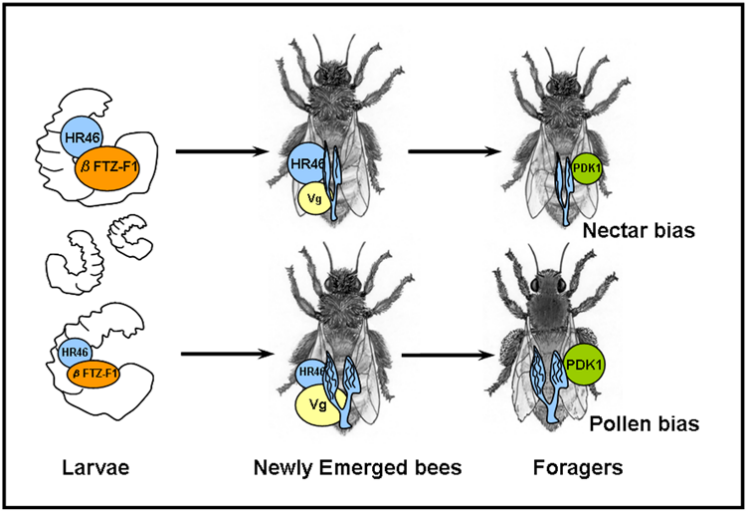
Genetic architecture of honey bee foraging behavior. *HR46* and *PDK1* can influence foraging behavioral decisions by acting during different life-stages and in different ways. As a putative affector of *βFTZ-*F1 activity, *HR46* may be part of the apoptotic signaling system that determines worker ovary size. Bees with large ovaries tend to have higher levels of *Vg* mRNA as young adults. *Vg* is a key behavioral affector gene that influences foraging onset and foraging bias: increased levels are associated with a bias toward early foraging onset and pollen bias. In foragers, ovary size is linked to *PDK1* gene activity, so that ovary size and *PDK1* mRNA levels both are higher in workers with a foraging bias toward pollen. In short, we propose that *HR46* acts in larvae to determine ovary size, which influences ovarian signaling in adult workers quantitatively and/or qualitatively, with effects on *Vg*, *PDK1*/IIS and foraging behavior.

Collectively, we have demonstrated a direct genetic link between the central reproductive trait, ovary size, and the pollen-hoarding syndrome of worker honey bees. In honey bees, ovary size is determined hormonally during larval development but it can remain a central endocrine player throughout life and may influence juvenile hormone and Vg (yolk protein) titers [40]. Our results suggest that *HR46* acts early during development to determine ovary size (Fig. 5). Further, although our findings at the level of mRNA transcript abundance do not exclude a developmental role also of *PDK1*, they are consistent with the idea that this gene is influenced by ovary size later in life where it can affect the life history trajectories of the adult bees (Fig. 5). The IIS pathway and the ecdysteroid cascade converge on the control of vitellogenesis and play major roles in orchestrating life history and reproduction in a variety of insects. Other associated gene networks may be involved and remain to be explored, but we propose that the two endocrine systems identified here provide a mechanistic basis for the RPGH playing a central role in the elaboration of complex insect societies and social behavior.

We believe RPGP captures fundamental principles and suggests future directions for broader research on the emergence of sociality. In female rats and humans, taste preference and food preferences change during the reproductive cycle. In some mammalian social species, for example naked mole-rats, meerkats and wolves, there are helpers who forage and take care of the nest instead of producing their own offspring [52], [53], [54]. It has been demonstrated that the neuronal network nodes of the ‘social behavior network’ contain receptors for sex hormones [55], which was a fundamental and evolutionarily conserved feature of the vertebrate brain [56]. Thus, the fundamental principles of the RGPH [4] could be applicable to vertebrate as well as invertebrate systems.

## Materials and Methods

### Effects of pln QTL on ovary size

Bi-directionally selected high- and low-pollen hoarding strains [9] were maintained by a circular inbreeding scheme with occasional outcrossing to unrelated stocks of similar phenotype at the University of California, Davis, US. The 23^rd^ generation of these strains served as parental generation for two reciprocal backcrosses performed in 2005. Hybrid queens were produced from one high and one low pollen-hoarding source colony by instrumental insemination. Both ovaries were dissected and the ovariole numbers were scored in 20 workers of each hybrid colony. Two queens derived from one hybrid colony were backcrossed to a single drone of the high- (high backcross, ‘HBC’) and of the low pollen-(low backcross, ‘LBC’) hoarding source colony [16]. Resulting worker offspring were transferred to an incubator just prior to emergence. We dissected both ovaries and successfully scored the number of ovarioles in 392 workers from the HBC and 393 workers from the LBC.

Initially, 95 workers with extreme phenotypes and the grandparental drone were selected from each backcross for genetic analyses. In the HBC, the sample size was doubled to confirm the effects found in the initial dataset. Whole genome DNA was extracted from head and thorax of each bee using a CTAB lysis and single phenol-chloroform extraction [14]. Each bee was genotyped in four single reactions at microsatellite loci [57] or SNP loci [58] that were closely linked to the *pln*-QTL and proved variable in the respective cross [59], [60], [61; SI Materials and Methods of QTL study and Table S2]. Data were evaluated by multi-way ANOVA (type I, fixed effects), based on the central limit theorem and the necessity to evaluate interaction terms between the four factors [17]. Non-parametric Mann-Whitney tests were used to reconfirm the main effects.

### Patterns of pln candidate gene expression level

Honey bees from the high- and low-pollen-hoarding strains [9] were maintained at Arizona State University and at the University of California, Davis, US. For each strain, third instar larvae, newly emerged adults, and mature foragers (returning from the field) were collected for qRT-PCR. Six sample bees were chosen randomly from each of two high- and two low strain colonies. Gene expression analysis was performed separately on the whole body of larvae and on the abdominal carcass and brain of adult honey bees for *PAR3*, *PDK1*, *PI3K 68D*, *IRS*, and *HR46* (primer sequences in SI Table S4).

Prior to RNA extraction, all tissues were flash-frozen in liquid nitrogen and stored at −80°C. RNA was extracted using RNeasy Mini Kit (Qiagen, Valencia, CA, US). Two-step qRT-PCR was used for expression analysis. First strand cDNA was generated using TaqMan Reverse transcription Reagents (ABI, Foster, CA, US). Real-time PCR was performed using QuantiTect SYBR®Green I (Qiagen, Valencia, CA, US) as described before [4]. *Actin* was used as active reference, and relative gene expression quantified by the comparative CT method [4].

### Associations between candidate gene expression level, ovary size and behavior

The 25^th^ generation high pollen-hoarding strain and low pollen-hoarding strain served as the parental generation for two additional reciprocal backcrosses at Arizona State University, US. Daughters were reared from the F_1_-hybrid queen mother and each queen was mated to a single male from the parental source of the hybrid queen. For the behavioral analyses, we used workers from one HBC colony with a mean ovariole count near the mid-parent value because pollen-hoarding behavior and the behavioral traits associated with the syndrome have been shown repeatedly to demonstrate directional dominance toward the low pollen hoarding traits [9], [14], [16], [17]. Therefore, the hybrid, low backcross, and low strain colonies were expected to be very similar in phenotype and low strain not informative for the backcross analyses. To study foraging behavior, newly emerged workers were marked with paint and introduced into a single-story Langstroth hive containing approximately 8,000 background bees (wild type). As soon as the experimental workers foraged, returning foragers were individually collected and pollen and sucrose loads were measured as described before [7], [20]. Data were evaluated by one-way ANOVA (fixed effects).

The same HBC was used as a source for newly emerged bees and foragers to compare ovary size to the expression level of candidate genes [21]. Gene expression analyses were performed in fat body with qRT-PCR (primers in SI Table S3). Fat body is the principle source of Vg, which is a key factor regulating foraging onset and foraging bias in adult honey bees. Fat body is the best target tissue to study for detecting the association between ovary size, behavior and candidate gene. Newly emerged bees were separated into two experimental groups based on ovary size: HO group with 22–38 ovarioles, and LO group with: 2–5 ovarioles. After dissection, tergites and adhering fat body were frozen in liquid nitrogen and kept at −80°C. RNA extraction and preparation, qRT-PCR and statistical analyses were performed as described above. Expression differences were contrasted to the high and low pollen-hoarding strain parental sources.

## Supporting Information

QTL Study S1 Materials and Methods of QTL study
(0.02 MB DOC)

Figure S1 Log transformation of the relative mRNA levels of PDK1 (Mean±s.e.m.) in the brain of high (blue bars) (n = 12) and low strain (red bars) bees (n = 12). The mRNA levels are measured as relative quantities (RQ). PDK1 shows no significant difference between high and low strain newly emerged bees and foragers in brain.
(0.11 MB TIF)

Figure S2 Log transformation of the relative mRNA levels of HR46 (Mean±s.e.m.) in the brain of high (blue bars) (n = 12) and low strain (red bars) bees (n = 12). The mRNA levels are measured as relative quantities (RQ). It shows there is no significant difference in HR46 expression between high and low strain newly emerged bees and foragers in brain.
(0.11 MB TIF)

Figure S3 Log transformation of the relative mRNA levels of PAR3 (Mean±s.e.m.) in the abdomen of high (blue bars) (n = 12) and low strain (red bars) bees (n = 12). The mRNA levels are measured as relative quantities (RQ). It shows there is no significant difference in PAR3 expression between high and low strain newly emerged bees and foragers.
(0.13 MB TIF)

Figure S4 Log transformation of the relative mRNA levels of PI3K (Mean±s.e.m.) in the abdomen of high (blue bars) (n = 12) and low strain (red bars) bees (n = 12). The mRNA levels are measured as relative quantities (RQ). It shows there is no significant difference in PI3K expression between high and low strain newly emerged bees and foragers.
(0.06 MB TIF)

Figure S5 Log transformation of the relative mRNA levels of IRS (Mean±s.e.m.) in the abdomen of high (blue bars) (n = 12) and low strain (red bars) bees (n = 12). The mRNA levels are measured as relative quantities (RQ). It shows there is no significant difference in IRS expression between high and low strain newly emerged bees and foragers.
(0.11 MB TIF)

Table S1 Statistical analysis results of PDK1 and HR46 in brain of high and low strain bees.
(0.03 MB DOC)

Table S2 Statistical analysis results of PAR3, PI3K and IRS in abdomen of high and low strain bees.
(0.04 MB DOC)

Table S3 Markers used to evaluate direct effects of behavioral QTL on worker ovary size.
(0.03 MB DOC)

Table S4 Primers of real-time PCR for the candidate genes.
(0.03 MB DOC)
