# Supplementary material for: PDK1 and HR46 Gene Homologs Tie Social Behavior to Ovary Signals
Source: PLoS One. 2009 Apr 2;4(4):e4899. doi: 10.1371/journal.pone.0004899 (PMC2659776; doi:10.1371/journal.pone.0004899)
Supplement: QTL Study S1 — Materials and Methods of QTL study (0.02 MB DOC) [file pone.0004899.s001.doc]

**Materials and Methods of QTL study**

The SNP marker was genotyped by MALDI-TOF mass spectrometry [59] through Sequenom® Inc. (San Diego, California). Microsatellite loci were PCR-amplified with a tailed-primer labeling method optimized for detection by a LiCor DNA analyzers [60, 61]. Individual reactions were carried out in 10 µl (1ng/µl template DNA, 125 nM forward primer, 250 nM reverse primer, 50 nM IRD700-labeled tail, 200 µM dNTPs, 0.5U Taq). The PCR thermoprofile used for all loci was a touchdown protocol [61]. Alleles were scored on a LiCor® 4200 automated DNA analyzer. Each individual genotype was scored twice to avoid genotyping errors.
