# Supplementary figures and images for: PDK1 and HR46 Gene Homologs Tie Social Behavior to Ovary Signals
Source: PLoS One. 2009 Apr 2;4(4):e4899. doi: 10.1371/journal.pone.0004899 (PMC2659776; doi:10.1371/journal.pone.0004899)

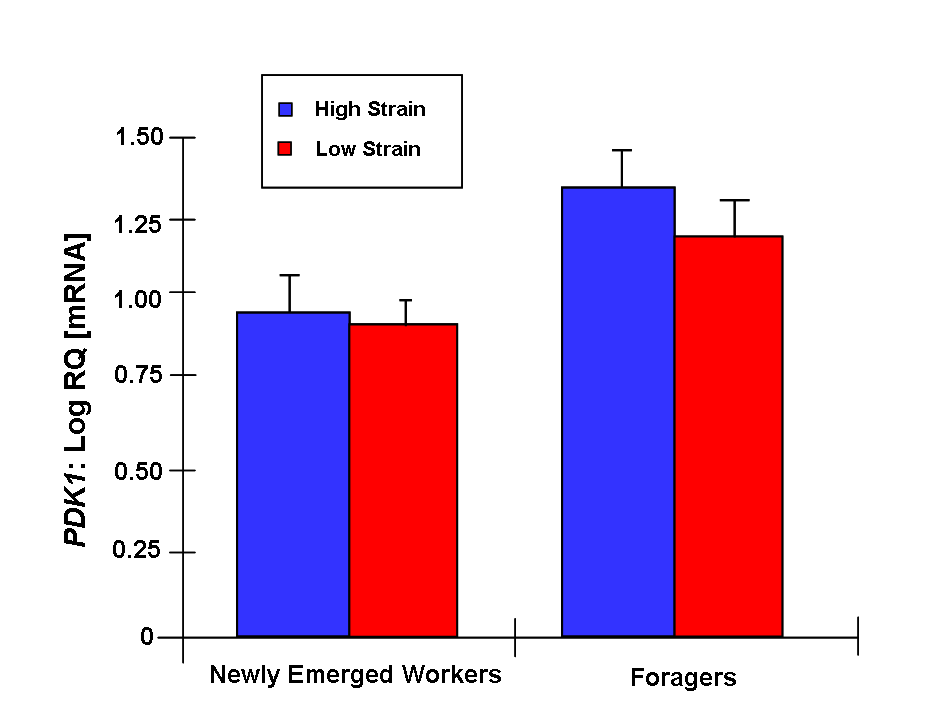

Supplement: Figure S1 — Log transformation of the relative mRNA levels of PDK1 (Mean±s.e.m.) in the brain of high (blue bars) (n = 12) and low strain (red bars) bees (n = 12). The mRNA levels are measured as relative quantities (RQ). PDK1 shows no significant difference between high and low strain newly emerged bees and foragers in brain. (0.11 MB TIF) [file pone.0004899.s002.tif]

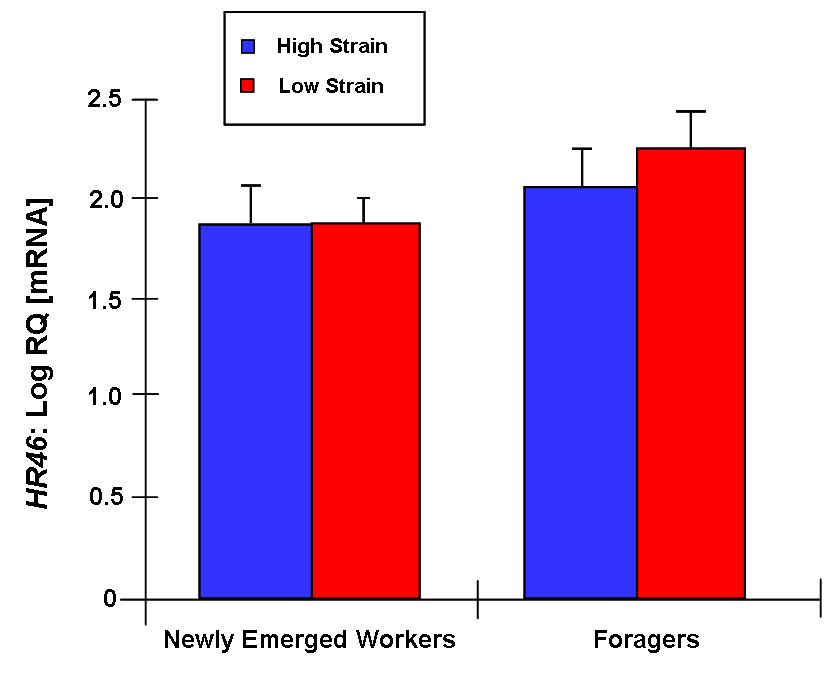

Supplement: Figure S2 — Log transformation of the relative mRNA levels of HR46 (Mean±s.e.m.) in the brain of high (blue bars) (n = 12) and low strain (red bars) bees (n = 12). The mRNA levels are measured as relative quantities (RQ). It shows there is no significant difference in HR46 expression between high and low strain newly emerged bees and foragers in brain. (0.11 MB TIF) [file pone.0004899.s003.tif]

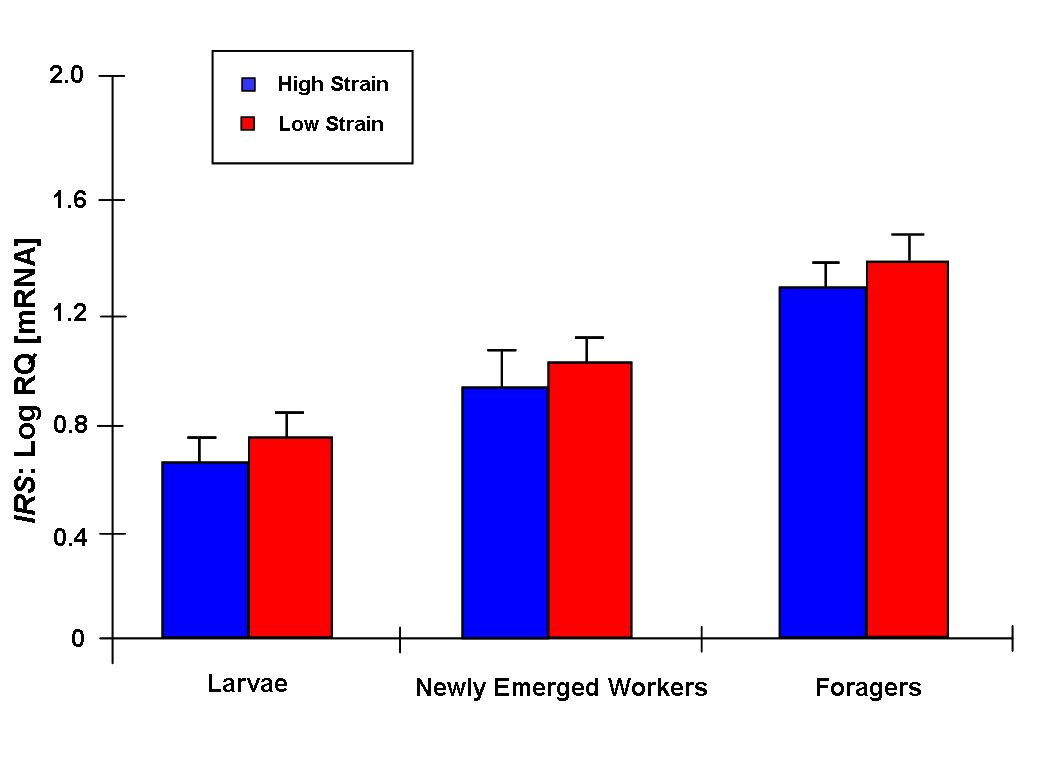

Supplement: Figure S5 — Log transformation of the relative mRNA levels of IRS (Mean±s.e.m.) in the abdomen of high (blue bars) (n = 12) and low strain (red bars) bees (n = 12). The mRNA levels are measured as relative quantities (RQ). It shows there is no significant difference in IRS expression between high and low strain newly emerged bees and foragers. (0.11 MB TIF) [file pone.0004899.s006.tif]
