# Supplementary material for: PDK1 and HR46 Gene Homologs Tie Social Behavior to Ovary Signals
Source: PLoS One. 2009 Apr 2;4(4):e4899. doi: 10.1371/journal.pone.0004899 (PMC2659776; doi:10.1371/journal.pone.0004899)
Supplement: Table S1 — Statistical analysis results of PDK1 and HR46 in brain of high and low strain bees. (0.03 MB DOC) [file pone.0004899.s007.doc]

**Table S1. Statistical analysis results of *PDK1* and *HR46* in brain of high and low strain bees.**

| **Target Gene** | **Bee Groups** | **t Value** | **df Value** | **p Value** |
| --- | --- | --- | --- | --- |
| ***PDK1*** | **Newly emerged bees between high and low strains** | **0.46** | **20** | **0.651** |
|  | **Foragers between high and low strains** | **1.12** | **19** | **0.278** |
| ***HR46*** | **Newly emerged bees between high and low strains** | **-0.07** | **15** | **0.943** |
|  | **Foragers between high and low strains** | **-8.05** | **13** | **0.409** |
