# Supplementary material for: PDK1 and HR46 Gene Homologs Tie Social Behavior to Ovary Signals
Source: PLoS One. 2009 Apr 2;4(4):e4899. doi: 10.1371/journal.pone.0004899 (PMC2659776; doi:10.1371/journal.pone.0004899)
Supplement: Table S2 — Statistical analysis results of PAR3, PI3K and IRS in abdomen of high and low strain bees. (0.04 MB DOC) [file pone.0004899.s008.doc]

**Table S2. Statistical analysis results of *PAR3, PI3K* and *IRS* in abdomen of high and low strain bees.**

| **Target Gene** | **Bee Groups** | **t Value** | **df Value** | **p Value** |
| --- | --- | --- | --- | --- |
| ***PAR3*** | **Larvae between high and low strains** | **0.47** | **21** | **0.643** |
|  | **Newly emerged bees between high and low strains** | **0.19** | **20** | **0.848** |
|  | **Foragers between high and low strains** | **-0.46** | **21** | **0.647** |
| ***PI3K*** | **Larvae between high and low strains** | **2.11** | **19** | **0.048** |
|  | **Newly emerged bees between high and low strains** | **-0.77** | **21** | **0.447** |
|  | **Foragers between high and low strains** | **-0.47** | **21** | **0.646** |
| ***IRS*** | **Larvae between high and low strains** | **-0.49** | **19** | **0.631** |
|  | **Newly emerged bees between high and low strains** | **-0.27** | **19** | **0.793** |
|  | **Foragers between high and low strains** | **2.00** | **21** | **0.059** |
