# Supplementary material for: PDK1 and HR46 Gene Homologs Tie Social Behavior to Ovary Signals
Source: PLoS One. 2009 Apr 2;4(4):e4899. doi: 10.1371/journal.pone.0004899 (PMC2659776; doi:10.1371/journal.pone.0004899)
Supplement: Table S3 — Markers used to evaluate direct effects of behavioral QTL on worker ovary size. (0.03 MB DOC) [file pone.0004899.s009.doc]

**Table S3. Markers used to evaluate direct effects of behavioral QTL on worker ovary size.**

| **Strains** | **QTL Marker** | | **Amel4.0 Scaffold** | **Position** |
| --- | --- | --- | --- | --- |
| **High backcross (HBC)** | ***Pln1*** | ***At003 microsatellite*** | **13.10** | **644,903 bp** |
|  | ***Pln2*** | **UN068 microsatellite** | **1.57** | **42,376 bp** |
|  | ***Pln3*** | **Ap016 microsatellite** | **1.24** | **1,079,340 bp** |
|  | ***Pln4*** | **Ap029 microsatellite** | **13.19** | **139,600 bp** |
| **Low backcross (LBC)** | ***Pln1*** | **Ap273 microsatellite** | **13.11** | **159,800 bp** |
|  | ***Pln2*** | **At110 microsatellite** | **1.59** | **246,536 bp** |
|  | ***Pln3*** | **Ap016 microsatellite** | **1.24** | **1,079,340 bp** |
|  | ***Pln4*** | **ahb4126 SNP** | **13.17** | **201,631 bp** |
