# Supplementary material for: PDK1 and HR46 Gene Homologs Tie Social Behavior to Ovary Signals
Source: PLoS One. 2009 Apr 2;4(4):e4899. doi: 10.1371/journal.pone.0004899 (PMC2659776; doi:10.1371/journal.pone.0004899)
Supplement: Table S4 — Primers of real-time PCR for the candidate genes. (0.03 MB DOC) [file pone.0004899.s010.doc]

**Table S4**. Primers of real-time PCR for the candidate genes.

| **Target Gene** | **GeneBank #** | **Forward Sequence** | **Reverse Sequence** |
| --- | --- | --- | --- |
| ***PAR3*** | **GB10346** | **5'CTAAACGAAACACGGACGAT3'** | **5'TCCGACATACTCTGCCTACC3'** |
| ***HR46*** | **GB10650** | **5'AGGTCGAGGACGAAGTTAGG3'** | **5'ATACCCGCCATTGAAAGGAT3'** |
| ***PI3K*** | **GB17429** | **5'TGAATTTGGCTTAACTGGAT3'** | **5'TTTCAACTGCTCGTTCGTAT3'** |
| ***PDK1*** | **GB15780** | **5'AGCTTCTCCACCGTTTACCT3'** | **5'ACTCCGTCTTCTTCTCCTTG3'** |
| ***IRS*** | **GB11037** | **5'AACCTTGTCGCTGCCCTTAG3'** | **5'GTTGGATGACTTTCGCTGGT3'** |
| ***Actin*** | **GB17681** | **5'TGCCAACACTGTCCTTTCTG-3'** | **5'AGAATTGACCCACCAATCCA3'** |
